# Distribution and Genotypic Landscape of Tick-Borne Encephalitis Virus in Ticks from Latvia from 2019 to 2023

**DOI:** 10.3390/pathogens14090950

**Published:** 2025-09-22

**Authors:** Lidia Chitimia-Dobler, Gerhard Dobler, Daniel Lang, Antra Bormane, Renate Ranka, Sabine Schaper, Zane Freimane, Dace Zavadska

**Affiliations:** 1 Fraunhofer Institute of Translational Medicine and Pharmacology ITMP, Institute of Immunology, Infection and Pandemic Research IIP, 80799 Munich, Germany; lidia.chitimia-dobler@itmp.fraunhofer.de; 2 Bundeswehr Institute of Microbiology, Neuherbergstrasse 11, 80937 Munich, Germany; daniellang@bundeswehr.org (D.L.); sabineschaper@bundeswehr.org (S.S.); 3 Parasitology Unit, Institute of Zoology, University of Hohenheim, 70599 Stuttgart, Germany; 4 Division of Infectious Diseases and Tropical Medicine, Medical Center of the University of LMU, 80802 Munich, Germany; 5 Unit Epidemiology, Centre for Disease Prevention and Control of Latvia, Infectious Diseases Surveillance and Immunization, 1005 Riga, Latvia; antra.bormane@spkc.gov.lv; 6 Latvian Biomedical Research and Study Centre, 1067 Riga, Latvia; renate.ranka@rsu.lv; 7 Faculty of Pharmacy, Riga Stradins University, 1007 Riga, Latvia; 8 Department of Pediatrics, Riga Stradins University, 1007 Riga, Latvia; zane.freimane@rsu.lv (Z.F.); dace.zavadska@rsu.lv (D.Z.); 9 Children’s Clinical University Hospital, 1004 Riga, Latvia

**Keywords:** tick-borne encephalitis, Latvia, phylogeny, geographic distribution, emergence

## Abstract

Ticks are important parasites of economic and public health because of their ability to transmit zoonotic diseases. Tick-borne encephalitis virus (TBEV), now officially *Orthoflavivirus encephalitidis*, is a Flavivirus with five main subtypes of which three, the European (TBEV-EU), the Siberian (TBEV-Sib), and the Far-Eastern subtypes (TBEV-FE), are supposedly circulate in Latvia. Several hard tick species are involved in TBEV circulation and transmission in nature. This study set out to describe the genetic landscape of TBEV subtypes in Latvian tick populations. In 2019 and 2021 to 2023, a total of 3026 ticks were collected in three Latvian regions, with *Ixodes ricinus* as the dominant species (2822 specimens), followed by *Ixodes persulcatus* (200 specimens) and *Dermacentor reticulatus* (4 specimens). Ticks were morphologically identified, pooled, and screened for TBEV RNA by RT-qPCR. TBEV of positive tick pools were isolated and genetically characterized by genome sequencing. Our study demonstrates the prevalence of two TBEV subtypes in Latvia with specific spatial patterning. In the sympatric Vidzeme region, these subtypes display a preferential species association of TBEV-EU with *Ixodes ricinus* and TBEV-Sib with *Ixodes persulcatus*. Phylogeographic analysis suggests independent introductions of multiple genotypes from both subtypes. Further investigations are necessary to better understand the natural transmission and the medical importance of these TBEVs.

## 1. Introduction

Tick-borne encephalitis (TBE) is the most important tick-borne viral disease in Europe and Asia. It is caused by *Orthoflavivirus encephalitidis* (formerly: tick-borne encephalitis virus (TBEV) (genus *Orthoflavivirus*, family Flaviviridae)) [1]. TBEV can be divided into at least five subtypes: the European, the Siberian, the Far-Eastern, the Baikalian, and the Himalayan subtypes [2,3]. In Europe, three of them are circulating—the European subtype, which is most prevalent in large parts of Europe and mainly associated with *Ixodes (I.) ricinus* and *Dermacentor (D.) reticulatus* ticks. The Siberian subtype, causing a clinical form, which was historically also named Russian Spring–Summer Encephalitis, is transmitted mainly by *I. persulcatus* and is prevalent in parts of Northern Europe and some areas in Southeastern Europe. Furthermore, there are some reports of the detection of TBEV-FE strains in small foci in Ukraine, Moldova, and Latvia [4,5,6].

TBEV is transmitted to humans by tick bite, although the possibility of infection by consuming unpasteurized dairy products has also been described [7]. In Europe, the notification rate of TBE has increased in recent years, and the risk areas for TBE infection are expanding [8]. Tick-borne encephalitis virus circulates in nature between ticks and small rodents. Unlike many other tick-borne pathogens, which are widespread in the tick population, TBEV circulates in very strictly defined, patchy natural foci. These natural foci in Central Europe are often found in small places the size of a soccer field [9]. Identification of these foci is therefore difficult, and many such areas remain undiscovered to date. Usually, microfoci are found based on data provided by TBE patients as an indication of potential locations where tick bites were acquired [10].

In Latvia, the ECDC TBE case definition has been used since 2012. TBE is prevalent throughout the whole country and is mandatorily reportable in Latvia. The incidence rates during the last 15 years have ranged from 5.9 to 15.7/100,000, instead of increasing vaccination rates in the Latvian population [11,12]. This variation in incidence in Latvia is poorly understood. However, the TBE case incidence rate depends on temporal, climatic, and geographic factors, vaccination coverage, as well as the abundance of suitable tick vectors in the environment and the population size of small mammals that are hosts of ticks and reservoirs for TBEV [13,14,15,16,17]. The exposure risk is a very significant factor in harboring tick-borne diseases. One well-known risk factor for a TBEV infection is spending over 10 h per week in forests [18].

So far, no detailed information on the distribution of TBEV in Latvia exists. More than 20 years ago, TBEV of all three subtypes was isolated from ticks from Latvia [6,19]. Since then, no detailed studies on the circulation and on the subtype of circulating TBEV have been conducted. A country-wide study conducted from 2017 to 2019 showed the presence of TBEV in different places, but without subtyping of the detected TBEV [20]. In another study, TBEV was detected in ticks collected from migratory birds of a high percentage [21]. Also here, unfortunately, no subtyping of the TBEV was performed.

Although Latvia is a TBE high-prevalence country and besides Ukraine, the only European country where the three major TBEV subtypes have been identified in the past, there are no actual data on the occurrence and prevalence of TBEV and its respective subtypes in Latvia. Also, the available data on the relative importance of specific vectors of TBEV in Latvia and the whole Baltic region are not comprehensive. The knowledge of the occurrence of TBEV subtypes is important to monitor the spread of the pathogen, the epidemiology of the disease, and prevention. Therefore, the current study was conducted to address the following objectives: (i) identify which TBEV subtypes are circulating in TBE microfoci; (ii) determine where the different subtypes are distributed; (iii) determine which tick species are transmitting which TBEV subtype; (iv) detect and subtype, sequence full genomes, and isolate the TBEV subtypes circulating in Latvia.

## 2. Materials and Methods

### 2.1. Tick Collection

**Figure 1:**
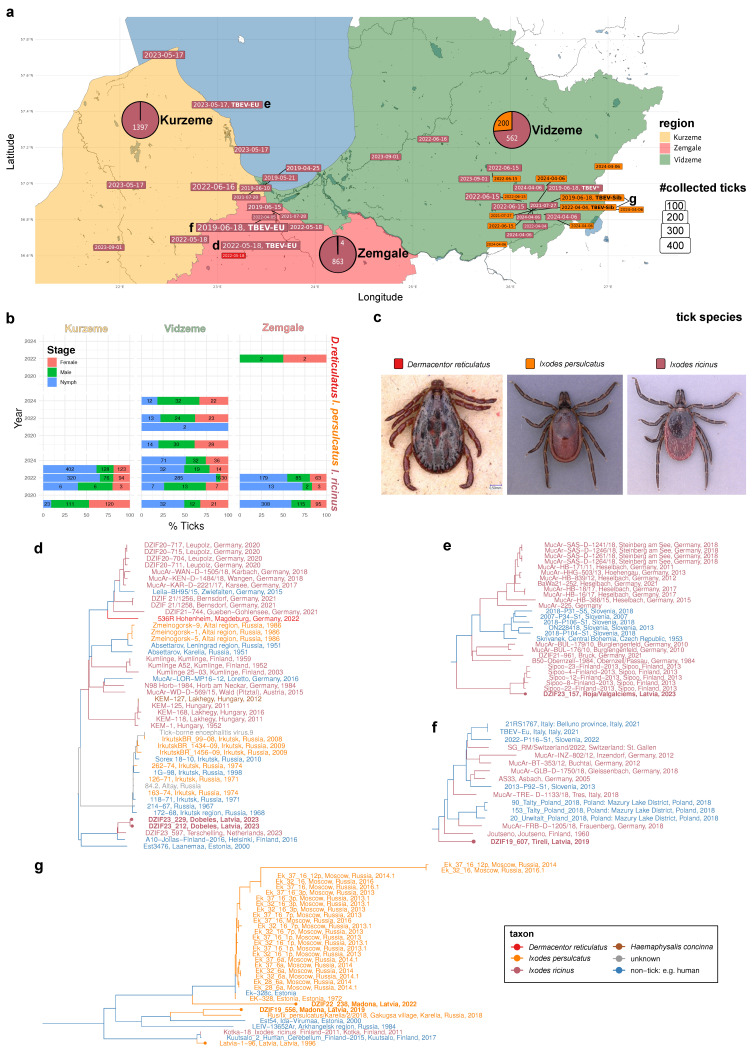
Distribution, genotypic landscape, and origins of tick-borne encephalitis viruses in Latvia. (**a**) Large-scale sampling of Latvian tick species with TBEV potential. Map depicting the three monitored Latvian regions: Kurzeme (light orange), Zemgale (light red), and Vidzeme (light green). Color-coded, dated boxes represent localization and timing of flagging-based sampling of three tick species: *D. reticulatus* (bright red), *I. ricinus* (dark red), and *I. persulcatus*. Box sizes are scaled according to the number of sampled ticks. TBEV-positive tick pools are emphasized by adding “TBEV” in bold font. If a full or partial genome could be sequenced, the respective TBEV subtype is displayed in bold font. Lower-case characters d-g near a dated box point to the subfigure displaying the phylogeny that comprises the respective TBEV isolate. Three pie charts depict the relative fraction of each tick species with respect to the total number of ticks sampled in each of the three Latvian regions. (**b**) Relative and absolute abundances of the juvenile and adult forms of the three tick species in the three Latvian regions. Stacked bar chart displaying the distribution of developmental stage or sex among the three sampled tick species in the three regions. (**c**) Example photographs of the sampled *D. reticulatus* (male), *I. persulcatus* (female), and *I. ricinus* (female) ticks. (**d**–**g**) Phylograms displaying the phylogenetic context of the Latvian TBEV isolates. Latvian isolates are highlighted with a tip point. The seven Latvian TBEV isolates sequenced in this study are displayed in bold font. Host taxon origins (tick species or non-tick origin) of the tips and inferred ancestral states are color-coded as depicted in the legend at the bottom right corner.

Ticks were collected from 2019 to 2024, in regions with high TBE incidence and in probable locations of infection, as reported by TBE patients, and in areas with tick surveillance data available from the Public Health Institute on TBEV in ticks (Figure 1, Table S1). Each place of collection was sampled for at least one hour by two to four samplers along the forest edges and in the forests, spanning respective areas in each sampling side of about 5000 sqm (estimated soccer field). Locations with TBEV-positive ticks were sampled several times, while a number of sampling locations (mostly TBEV-negative) were only sampled once (details are in Supplementary Table S1). Ticks were collected by dragging/flagging, stored in 50 mL Falcon tubes, and kept alive until morphological identification and sorting at the Bundeswehr Institute of Microbiology in Munich.

### 2.2. Molecular Detection

Ticks were tested individually or in pools of 2 to 10 specimens/pool according to collection site, tick species, and life stage. After sorting, the ticks were crushed three times at a speed of 6.5 rpm for 30 s in the Fast Prep Savant FP120 tissue lyser (Bio101, Vista, CA, USA) in 1 mL of Minimum Essential Medium (MEM, Invitrogen, Karlsruhe, Germany) containing an antibiotic–antimycotic solution (ABAM, Invitrogen) [22]. The nucleic acid was extracted using the MagNA Pure LC Total Nucleic Acid Kit (Roche, Mannheim, Germany) in the MagNA Pure LC instrument (Roche, Mannheim, Germany), according to the manufacturer’s instructions, using 200 μL of the tick homogenate supernatant. The extracted samples were tested for the presence of TBEV using real-time RT-PCR (RT-qPCR), targeting the 3′-noncoding region of the viral genome and 5 μL of the eluted RNA [23]. The homogenates were kept at −80 °C until they were used undiluted and in a dilution of 1:5 and 1:25 for virus isolation. For virus isolation, a 500 μL aliquot of the diluted supernatants of the RT-qPCR-positive tick pools was added to an 80% confluent cell culture of A549 cells (human lung carcinoma cells, German Collection of Microorganisms and Cell Cultures, Braunschweig). After 1 h of incubation at 37 °C, the supernatant was decanted and the cells were washed three times with MEM containing ABAM. In total, 5 mL of MEM containing 10-fold concentrated ABAM and 3% fetal calf serum were added. Cells were incubated for up to 7 days at 37 °C and observed daily for the occurrence of cytopathogenic effect (cpe) [22]. In the case of more than 50% cpe, the supernatant was taken and tested by RT-qPCR for TBEV as described. In the case of no cpe, culture supernatant was taken after 7 days of incubation and tested for growth of TBEV by RT-qPCR. No subcultures were conducted. From the isolated TBEV strains, whole genomes were sequenced for confirmation as described [24]. It was assumed that only one tick specimen in the pool was infected if the pool tested positive, and the minimal infection rate (MIR) was estimated.

### 2.3. Genome Sequencing and Phylogenetic Inference

Targeted enrichment short-read sequencing of six TBEV-positive samples was performed using the hybridization capture with the Comprehensive Viral Research Panel™ on the Illumina MiSeq™ platform.

Genome sequences were derived via the gramtools v1.10.0 [25] and freebayes v1.3.6 [26] software. The resulting sequences were compiled with the most closely related TBEV sequences obtained from the NCBI Virus database, determined by screening with minimap2 [27]. A bootstrapped maximum-likelihood phylogeny of resulting genome sequences was inferred with the GTR model implemented in the FastTree Version 2.1.11 Double precision [28] software based on a multiple genome alignment constructed with the MAFFT v7.520 [29] software. The Latvian TBEV isolates were traced in the resulting tree topology to define well-supported, evolutionary clades, which are presented in Figure 1d–g. Supplementary Figure S3 d–g depict cladograms of the chosen tree clades presented in Figure 1, with bootstrap support values and NCBI accession numbers.

## 3. Results

### 3.1. Prevalence of Three Tick Species with TBEV Transmission Potential in Latvia

Over five years in the months between April and September, we collected a total of 3026 ticks by flagging at 23 different locations in three Latvian regions (Figure 1a–c; Supplementary Table S1). Three tick species belonging to two genera were identified. With 2822 specimens, comprising 594 males, 582 females, and 1646 nymphs, *I. ricinus* was the dominant species and found in all regions studied. *I. persulcatus* ticks were found only at eight sites in the Vidzeme region. With 200 specimens, comprising 86 males, 73 females, and 41 nymphs, it seems to be geographically more restricted than *I. ricinus* (Figure 1b).

**Figure 2:**
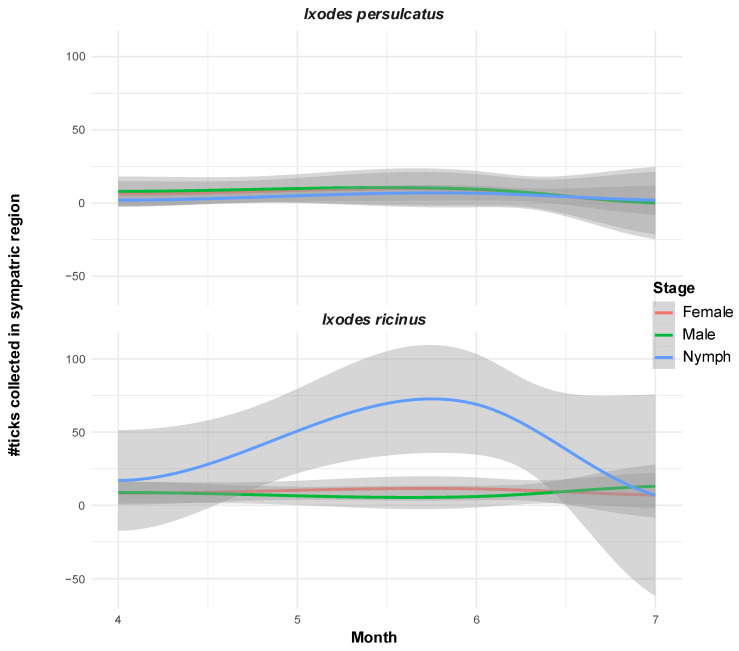
Disjunct periods of nymph activity of the two *Ixodes* species in sympatric locations in Vidzeme. Loess-smoothed curves of conditional means (colored lines) with confidence intervals (gray areas) of number of ticks per monitored developmental stage sampled between months of April and July between 2019 and 2023 at Vidzeme locations where both *Ixodes* species were found at least once.

While *I. ricinus* was found in all months sampled, the activity of *I. persulcatus* was found mainly in the months of April, May, and June (except two nymphs which were found at the end of July 2022 in Madona, Vidzeme (Figure 2). *D. reticulatus*, for which only four adults (two males and two females) were sampled, could be found exclusively in the location of Dobele (Zemgale region).

### 3.2. Prevalence of TBEV in the Monitored Regions

A total of six positive tick pools were detected (6/3026 ticks positive; 0.2% overall). Regarding TBEV-EU, 3/2822 ticks (0.1%) were found positive. All TBEV-EU-positive ticks belonged to the species *I. ricinus*. They were detected in the three locations, Tirele, Dobele, and Roia/Valgalciems, in the districts of Zemgale and Kurzeme (Supplementary Table S1). The TBEV-EU-positive ticks were sampled in June 2019, May 2022, and May 2023. The ct values of the semi-quantitative rtPCR used ranged between 24.56, 24.05, 31.13, and 28.67 (average, 27.10). The TBEV strains of all three positive tick pools were successively isolated in cell culture, and the whole genomes were sequenced.

In two of the PCR-positive tick pools, TBEV-Sib was identified. The overall prevalence rate was 2/200 (1%) and, therefore, five times higher than for TBEV-EU. Both TBEV-Sib-positive ticks belonged to the tick species *I. persulcatus* and were collected in the location of Madona in the district of Vidzeme. The ticks were collected in June 2019 and May 2022. The ct values of the two TBEV-Sib-positive tick pools were 17.50 and 23.15, both lower than the TBEV-EU-positive pools (average ct value, 20.36). The TBEV-Sib strains of the two pools could also be successfully isolated in cell culture, and the whole genome could be sequenced.

One positive tick pool, collected in Madona/Vidzeme in June 2019 from *I. ricinus,* could not be subtyped or isolated. The ct value was 32.86 and we were not able to produce any sequence for subtyping. This TBEV-positive material was not included in any further analysis.

### 3.3. Latvian Isolates from Two TBEV Subtypes Cluster in Six Distinct Evolutionary Clades

We performed targeted enrichment sequencing of the seven TBEV-positive samples (Figure 1a). Consistent with their respective RT-qPCR C_t_- values, whereas one of the samples yielded insufficient sequence information (Madona, 19 June 2019, *I. ricinus*, ct = 32.86), six of the samples could be genotyped to infer five full-length (ct min = 17.5; ct max = 28.67) and one partial (>6kb; Madona, 4 April 2022, *I. persulcatus*, ct = 23.15) genome sequence.

Combining the novel isolates with the existing whole genome of the TBEV strain *Latvia 1-96* (GU183382) [6], we subsequently traced the genetic origins of the seven genomic Latvian TBEV isolates by identifying the 115 most similar TBEV genomes followed by phylogenetic inference. Analysis of the resulting genome-wide phylogenies revealed six subclades from two TBEV subtypes harboring Latvian genotypes (Figure 1d–g).

In Kurzeme and Zemgale, all isolates fall within the European TBEV subtype. The two Dobele isolates (DZIF23_212; DZIF23_229) from the Zemgale region cluster with three TBEV-EU genotypes isolated from *I. ricinus* and two human cases from Finland and Estonia (Figure 1d). The nearby Tīreļi isolate (DZIF23_157), about 36 km away from Dobele, belongs to a distinct phylogenetic lineage grouping basal to another TBEV-EU clade and is closest to a 1960 Finnish isolate from South Karelia (Figure 1f). The northmost Latvian TBEV isolate from Roja/Valgalciems in Kurzeme (DZIF19_607) groups with five South Finnish TBEV-EU isolates from *I. ricinus* ticks in Sipoo (Figure 1e).

The two *I. persulcatus*-borne isolates belong to the evolutionary subtype of the Siberian TBEV subtype. Strikingly, the two Madona isolates do not fall into the same TBEV-Sib subclade and cluster independently of the previously reported human isolate Latvia 1-96, which robustly groups with two Finnish genotypes (Figure 1g). The 2022 Madona isolate (DZIF22_238) groups basal to, but is clearly distinct from, a group of TBEV-Sib viruses also comprising the 1972 Estonian *I. persulcatus* isolate *EK-328* [30]. The 2019 Madona isolate (DZIF19_556), on the other hand, seems to belong to a distinct genotypic lineage clustering closely with a North Estonian and a Russian–Karelian *I. persulcatus*-borne isolate.

## 4. Discussion

Latvia is, besides Ukraine, the only known European country where the three major TBEV subtypes have been identified in the past [19]. In the last 20 years, no information on the presence and the geographical distribution of TBEV subtypes in Latvia has been available. The results of our study confirm the presence of two TBEV subtypes, the TBE-EU and the TBEV-Sib. Furthermore, three tick species of two tick genera could be identified, *I. ricinus*, *I. persulcatus,* and *D. reticulatus*. Our tick flagging survey of 23 locations in Latvia, spanning a period of five years, revealed spatial distribution patterns of the three tick species that are highly consistent with previous large-scale studies [20,31,32] (Supplementary Figure S1). While *I. ricinus* displays clear overall prevalence in all three sampled Latvian regions (91% of total sites), the spatial distribution of *I. persulcatus* in our study is limited to the Eastern part of Latvia (Vidzeme region; 34% of total sites). During our sampling period, *D. reticulatus* was absent from the Northern parts of Latvia and was sampled only in Dobele in the Zemgale region (4% of sites). While previous studies have reported a slightly more widespread sampling of this tick species, the spatial distribution pattern limiting its distribution in our study to the southern part of Latvia is highly consistent. While all stages of *I. ricinus* could be detected from April to September, adult *I. persulcatus* was mainly active early in the year from April to June (two active nymphs were found in July; Figure 2). Not much data is available about the phenology and activity maxima of the three species in Latvia, and our sampling was not directed to comprehensively sample the species’ activity periods. Therefore, only limited insights on the phenology of the three tick species can be drawn from our data.

Consistent with previous analyses primarily limited to the TBEV E gene, our phylogenetic analysis of the geography, host taxonomy, and full-genome sequence of the sampled TBEV genotypes in Latvia clearly demonstrates the existence of two TBEV subtypes, the European as well as the Siberian subtypes of tick-borne encephalitis virus [6]. TBEV-EU was detected only in *I. ricinus*. As earlier data show a country-wide distribution of this tick species, it is reasonable to speculate that TBEV-EU may have a countrywide distribution [20]. Our data could identify three different genetic lineages of TBEV-EU. All three TBEV-EU viruses from Latvia show phylogenetic relations to three genetically clearly separated TBEV-EU genotypes originating in Finland. Thus, we find evidence for multiple independent introductions of distinct TBEV genotypes and their respective establishment as natural foci in different ecological niches in Latvia. These data argue for independent introductions mainly in a north–south direction. Unfortunately, we do not have sufficient data on TBEV from Estonia, between Finland and Latvia, to dissect if these introductions were continuous by ground animals, discontinuous by bird migration, or a combination of both.

TBEV-Sib was only detected in *I. persulcatus* in one specific area (Madona) in the Vidzeme region. Earlier data showed a greater area of distribution of the Taiga tick in parts of Eastern and Northern Latvia (Supplementary Figure S1). Strikingly, the two TBE-Sib strains isolated from ticks in Madona stem from phylogenetically related but distinct clades. Similarly to the two Zemgale TBEV-EU genotypes, phylogenetic evidence suggests independent introductions of two distinct TBEV-Sib genotypes at the same location. One strain (DZIF-22-238) belongs to the genotypic lineage of EK-328, a strain isolated from a pool of *I. persulcatus* ticks in 1972 in Estonia (PRJNA473492). The other isolated strain (DZIF-19-556) can be traced to a distinct lineage likely originating in the Northwestern District of Russia, e.g., Karelia and Arkhangelsk. Both strains are distinct from the TBEV-Sib strain Latvia 1-96, isolated in 1996 [19], which has the closest genetic relationship to strains from Finland. These data now show that TBEV-Sib was introduced to Latvia from different geographical directions and on different occasions. But again, we unfortunately do not have sufficient numbers of TBEV strains or sequences to obtain a more complete picture of the introduction histories and migration routes of these viruses into the Baltic region. The detection of the two recent TBEV-Sib strains in one location and the negative testing of many Taiga ticks from different regions in Latvia argues for a similar microfocal occurrence of TBEV-Sib as found in Central Europe for TBEV-EU [24,33]. More detailed studies on the structure of TBEV-Sib natural foci are not available. One Finnish study found TBEV on a Finnish island in *I. ricinus* [34]. More detailed phylogenetic analyses of TBEV-Sib in Russia also imply a microstructural occurrence and distribution of this virus subtype in nature [35].

All collected *D. reticulatus* ticks tested negative. *D. reticulatus* only recently came into focus as an additional vector of TBEV, extending its distribution from Lithuania to Latvia. It is unclear how long this tick species has been present in Latvia [32]. However, in parts of Poland and Germany, this tick species seems to play an important role in the natural transmission cycle of the TBE virus [22,36]. Our negative results do not exclude a substantial role of *D. reticulatus* in the natural transmission cycle of TBEV, at least in a local geographical range, as only a few ticks of this species were sampled from one single location. In an earlier study, TBEV was found in *D. reticulatus* ticks at a similar prevalence as in *I. ricinus* and *I. persulcatus* [20].

Our results also do not unambiguously exclude the occurrence of the third TBEV subtype, TBEV-FE, found in Latvia in 1996 [19]. Our results indicate a microfocal structure of TBEV foci in Latvia, similar to the small structures found in Central Europe [24]. Therefore, in some Latvian areas, *I. persulcatus* might also host TBEV-FE strains which have not been detected yet.

The increased genomic resolution, taxon sampling, and metadata now enable us to distinguish between the phylogenetic origins of the identified TBEV microfoci as well as to shed light on the potential routes by which these genotypes spread and arrived in the Baltics. While the TBEV-Sib subtype in Latvia so far has been found only in Vidzeme, the northeastern part of Latvia, microfoci of the TBEV-EU have been found to be more widely spread. The phylogeography analysis using the full-genomic data of the TBEV isolates, as well as the distribution of the tick vectors display three distinct patterns: The Roja isolate belongs to a Central European clade, with potential Bohemian heritage; Dobele isolates have high diversity, can be traced to TBEV-EU lineages dating back to 1950/60, are still circulating there today, and spread to inland North and Central Europe, having direct sister taxa along the coasts of the North and Baltic sea. It is tempting to speculate whether these specific patterns may be found in distinct biological routes of transmission (e.g., co-feeding of distinct tick species on migrating and ground breeding water fowl in coastal or freshwater habitats rich in bird species).

Despite their distinct and diverse genotypic origins, all six TBEV isolates share a common pattern with respect to their biogeography and host taxonomy—isolates from either human cases or ticks in Baltic states, and some are also found in the sympatric regions of *I. ricinus* and *I. persulcatus*. In all cases, sister taxa of Latvian TBEV comprise isolates collected in Finland and Estonia, and except for the Tireli isolate, the genotypic lineage can be traced to older Russian isolates from the Karelia region.

The sympatric occurrence of TBEV-EU and TBEV-Sib in Latvia may make it possible for TBEV-Sib strains to switch to *I. ricinus* and TBEV-EU to *I. persulcatus* [37]. Wang et al. predicted a much more extensive geographic distribution potential for I. persulcatus than is indicated by the actual reported collections [38]. Furthermore, with progressing climate change, habitats of both ticks may align even more. In their study, Wang et al. also demonstrated a large overlap of host networks for juvenile and adult life stages of both species, *I. ricinus* and *I. persulcatus* (Supplementary Figure S2). Together with the overlap of habitats, this increases the probability of spillover of formerly species-specific TBEV subtypes to new tick vector species. Earlier works clearly showed that TBEV-Sib may circulate in *I. ricinus* [34]. The co-occurrence of both TBEV subtypes and their respective major vectors makes switching events possible and therefore bears the risk that the TBEV-Sib subtype may adapt to *I. ricinus* and then may spread further south into Central Europe. The exact adaptation mechanisms are not known so far, and therefore, the sympatric zones in the Baltics may act as areas of host transition events. Another risk in areas of cocirculation of both TBEV subtypes is the recombination of genomes from different subtypes. A study identified the Baikalian subtype with highly pathogenic properties as a recombination of TBEV-Sib and TBEV-FE [19,39].

There is some scientific discussion of how TBEV-Sib reached the Baltics and Finland. In their earlier research, Kovalev et al. postulated three sub-lineages in the Baltic clade of the TBEV-Sib [35]. This work was based on the analysis of clusters using the E genes of the available strain data. There, the authors state that according to their calculations, the TBEV-Sib Baltic developed some 300 years ago. In some earlier analyses, Kovalev et al. show an association of the spread of TBEV-Sib to the European part of Russia and further to the Baltics and Finland with the construction of the Trans-Siberian Highway and the Trans-Siberian Railway [40,41]. In a recent study, Tkachev et al. distinguish five genetic lineages within TBEV-Sib: among the Baltic lineage, a Vasilchenko lineage, a Zausaev lineage, an Obskaya lineage, and a Bosnian lineage [42]. Our two isolates of the Siberian subtype fall into two genetic clades within the Baltic lineage: one strain is more closely related to an Estonian strain (EK-328), and one isolate is more closely related to a Karelian strain (Rus/*Ixodes persulcatus*/2/2018). These data argue for multiple and independent importations of TBEV strains from different directions in Latvia. A similar phenomenon for the introduction of TBEV has been found for TBEV-EU in different parts of Central Europe [24].

## Data Availability

All geographical data are made publicly available in the Supplementary Material. The whole-genome sequences will be submitted to the NCBI nucleotide database.

## Supplementary Materials

The following supporting information can be downloaded at https://www.mdpi.com/article/10.3390/pathogens14090950/s1. Figure S1: Geographical distribution of collected ticks and identified TBEV in Latvia in the past; Figure S2: Tick–host relations, particular and common hosts of *Ixodes ricinus* and *Ixodes persulcatus*; Figure S3: High resolution phylograms with bootstraps of the Latvian TBEV isolates; Table S1: summary of tick collections mentioned in the study.

## Abbreviations

The following abbreviations are used in the manuscript:

TBE	Tick-borne encephalitis
TBEV	Tick-borne encephalitis virus
TBEV-EU	Tick-borne encephalitis virus, European subtype
TBEV-Sib	Tick-borne encephalitis, Siberian subtype
TBEV-FE	Tick-borne encephalitis, Far-Eastern subtype
ECDC	European Center for Disease Control
MEM	Minimal Essential Medium
ABAM	Antibiotic–Antimycotic solution
RNA	Ribonucleic acid
RT-PCR	Reverse transcriptase polymerase chain reaction
RT-qPCR	Quantitative reverse transcriptase polymerase chain reaction
MIR	Minimal infection rate
NCBI	National Center for Biotechnology Information
GTR	Generalized time-reversible
MAFFT	Multiple alignment program for amino acid or nucleotide sequences
I.	Ixodes
D.	Dermacentor
Ct value	Cycle threshold value
