# Supplementary material for: Distribution and Genotypic Landscape of Tick-Borne Encephalitis Virus in Ticks from Latvia from 2019 to 2023
Source: Pathogens. 2025 Sep 22;14(9):950. doi: 10.3390/pathogens14090950 (PMC12472722; doi:10.3390/pathogens14090950)
Supplement: Supplementary file 1 [file pathogens-14-00950-s001.zip › Supplementary_Figure S1.pdf]

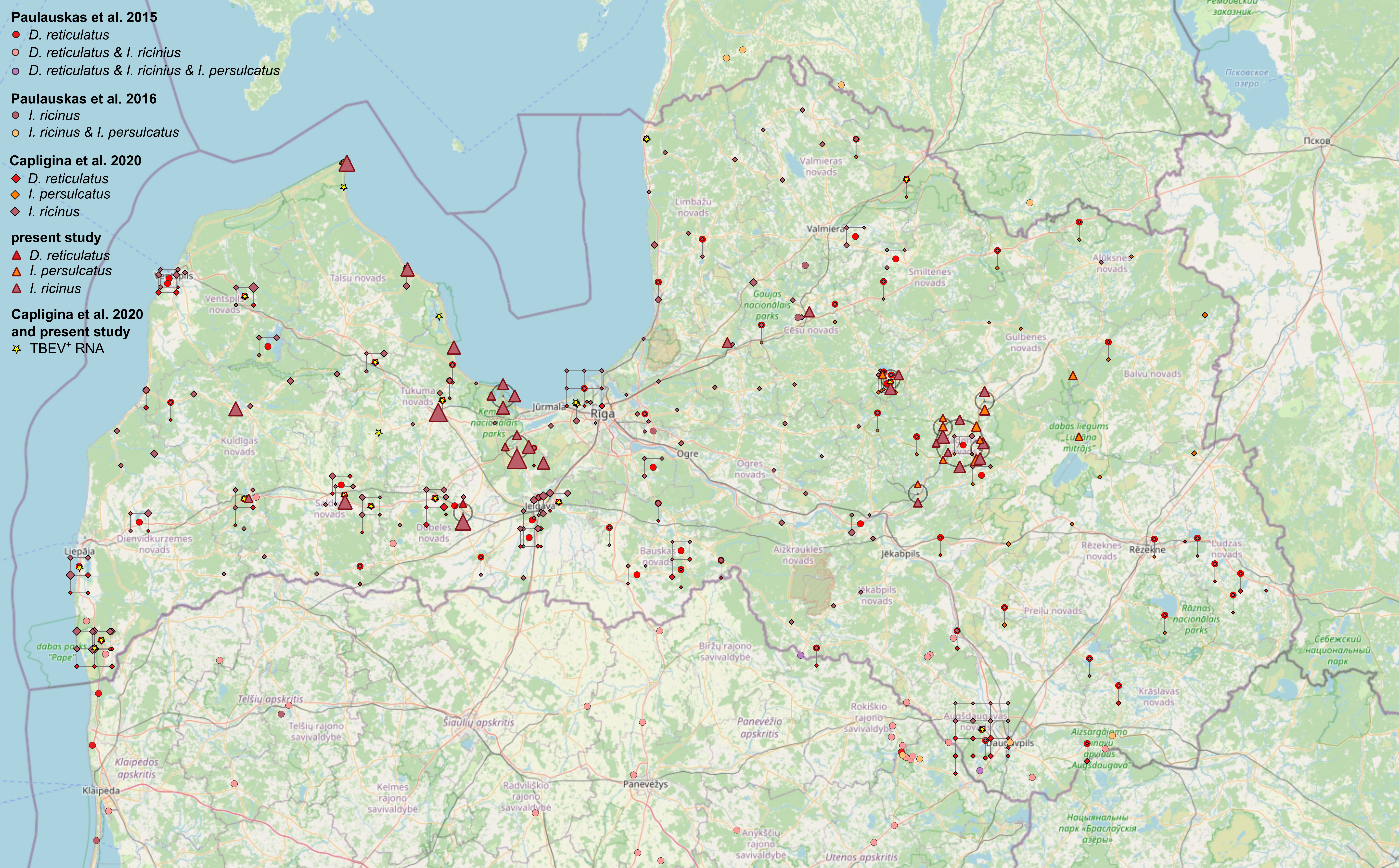
Paulauskas et al. 2015
D. reticulatus
D. reticulatus & I. ricinius
D. reticulatus & I. ricinius & I. persulcatus
Paulauskas et al. 2016
I. ricinus
I. ricinus & I. persulcatus
Capligina et al. 2020
D. reticulatus
I. persulcatus
I. ricinus
present study
D. reticulatus
I. persulcatus
I. ricinus
Capligina et al. 2020 and present study
TBEV⁺ RNA
Valmieras novads
Limbažu novads
Valmiera
Smiltenes novads
Alūksnes novads
Gaujas nacionālais parks
Cēsu novads
Gulbenes novads
Balvu novads
Talsu novads
Ventspils novads
Tukuma novads
Jūrmala
Rīga
Ogre
Ogres novads
dabas liegums „Lubāna mitrājs"
Kuldīgas novads
Saldus novads
Dobeles novads
Jelgava
Bauskas novads
Aizkraukles novads
Jēkabpils
Jēkabpils novads
Rēzeknes novads
Rēzekne
Ludzas novads
Dienvidkurzemes novads
Liepāja
Preiļu novads
Rāznas nacionālais parks
Biržų rajono savivaldybė
Krāslavas novads
Telšių apskritis
Telšių rajono savivaldybė
Šiaulių apskritis
Panevėžio apskritis
Rokiškio rajono savivaldybė
Augšdaugavas novads
Daugavpils
Klaipėdos apskritis
Kelmės rajono savivaldybė
Radviliškio rajono savivaldybė
Panevėžys
Anykščių rajono savivaldybė
Utenos apskritis
Klaipėda
Псков
Псковское озеро
Себежский национальный парк
Нацыянальны парк «Браслаўскія азёры»
