# Supplementary material for: Distribution and Genotypic Landscape of Tick-Borne Encephalitis Virus in Ticks from Latvia from 2019 to 2023
Source: Pathogens. 2025 Sep 22;14(9):950. doi: 10.3390/pathogens14090950 (PMC12472722; doi:10.3390/pathogens14090950)

Figure S3 d supplementary cladogram with bootstrap support values

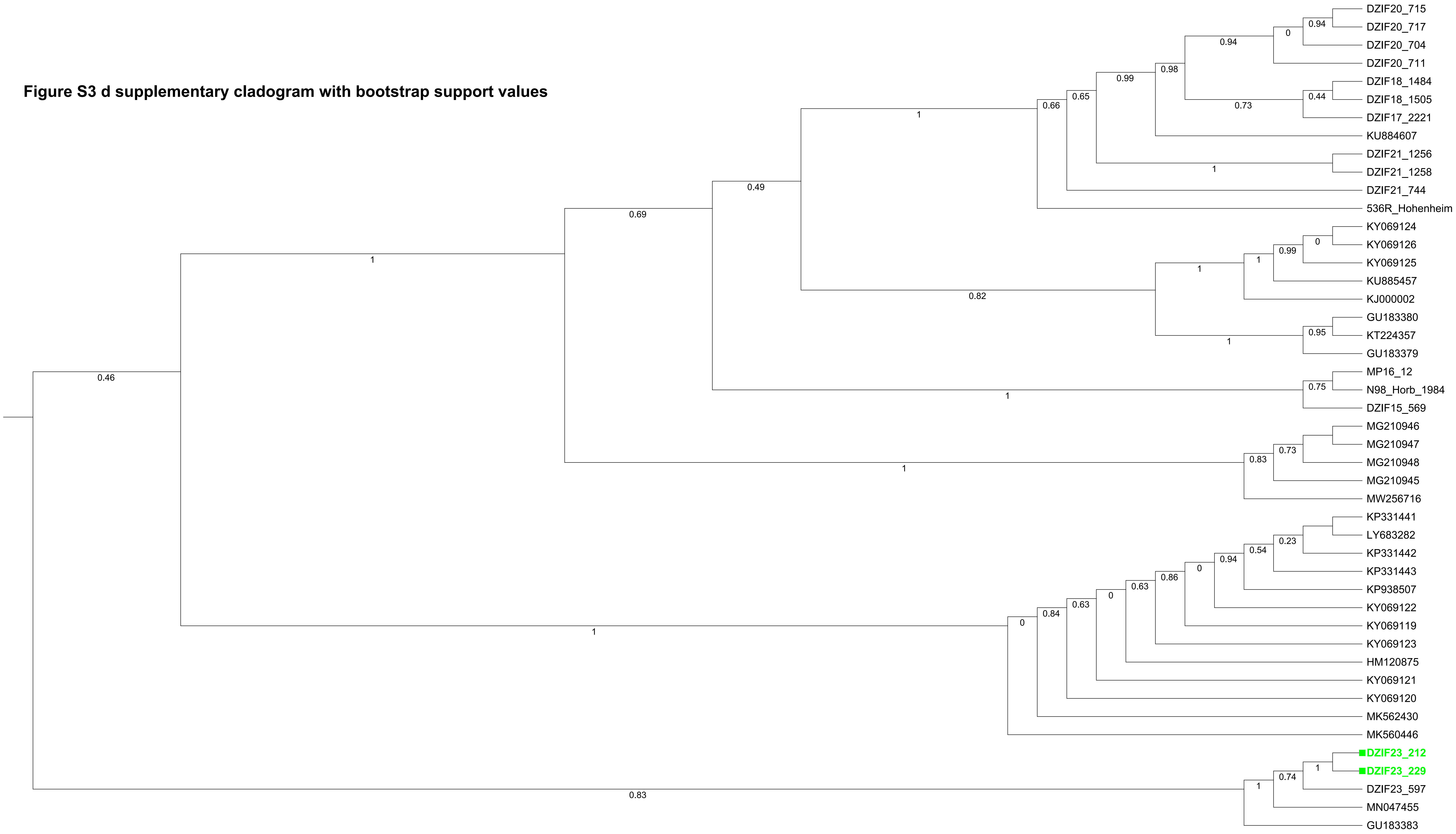

Figure S3 e supplementary cladogram with bootstrap support values

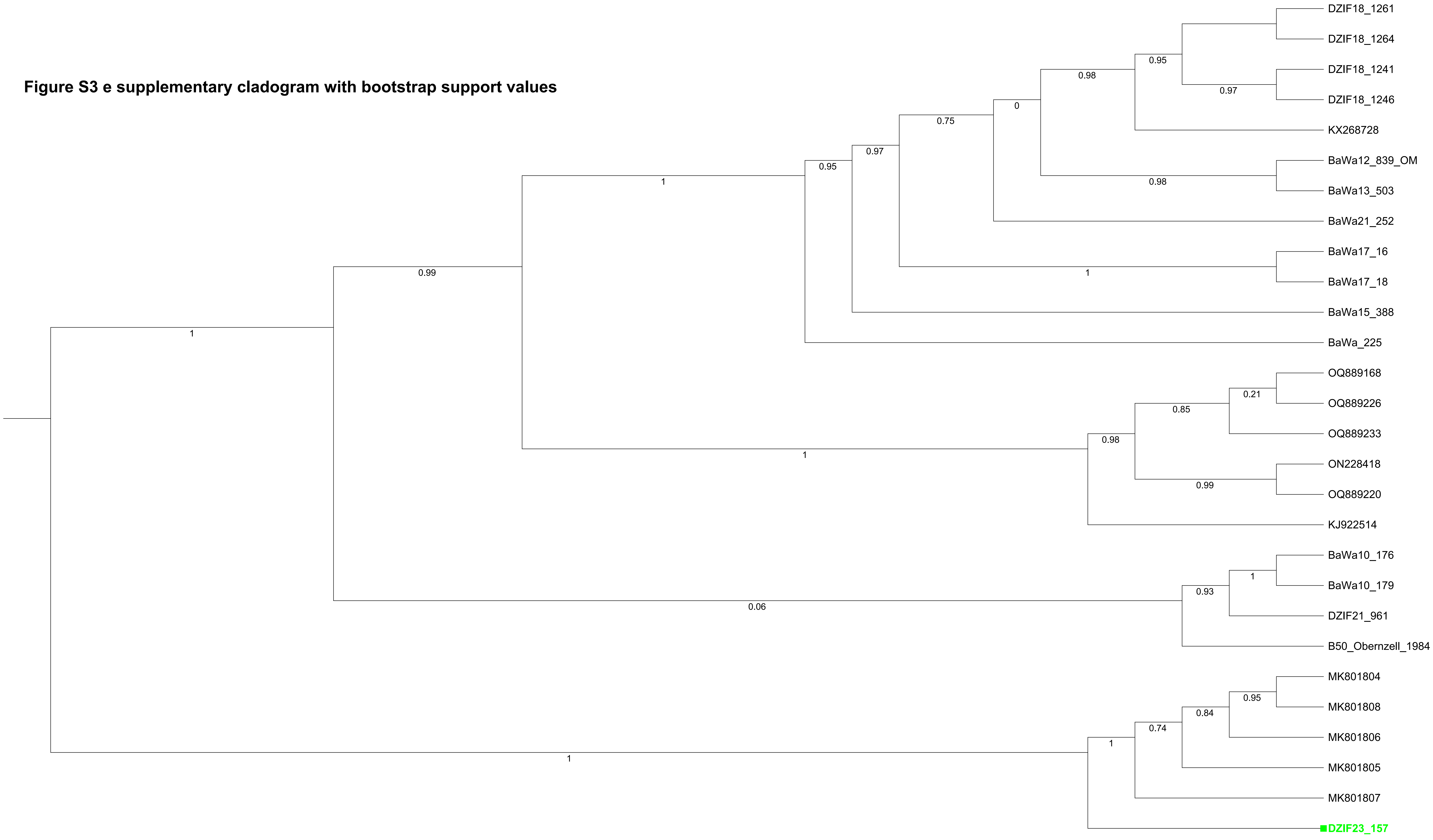

Figure S3 f supplementary cladogram with bootstrap support values

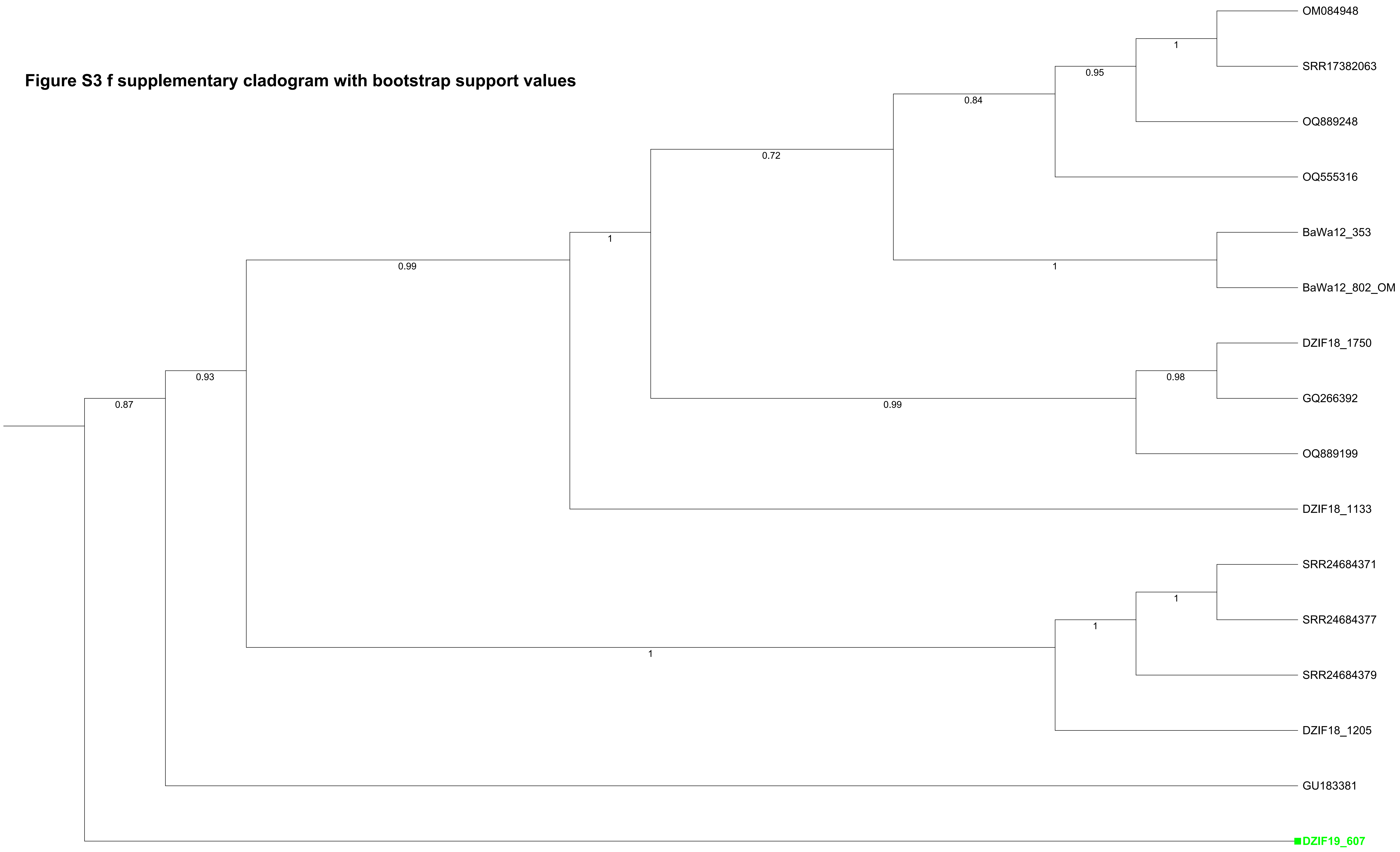

Figure S3 g supplementary cladogram with bootstrap support values

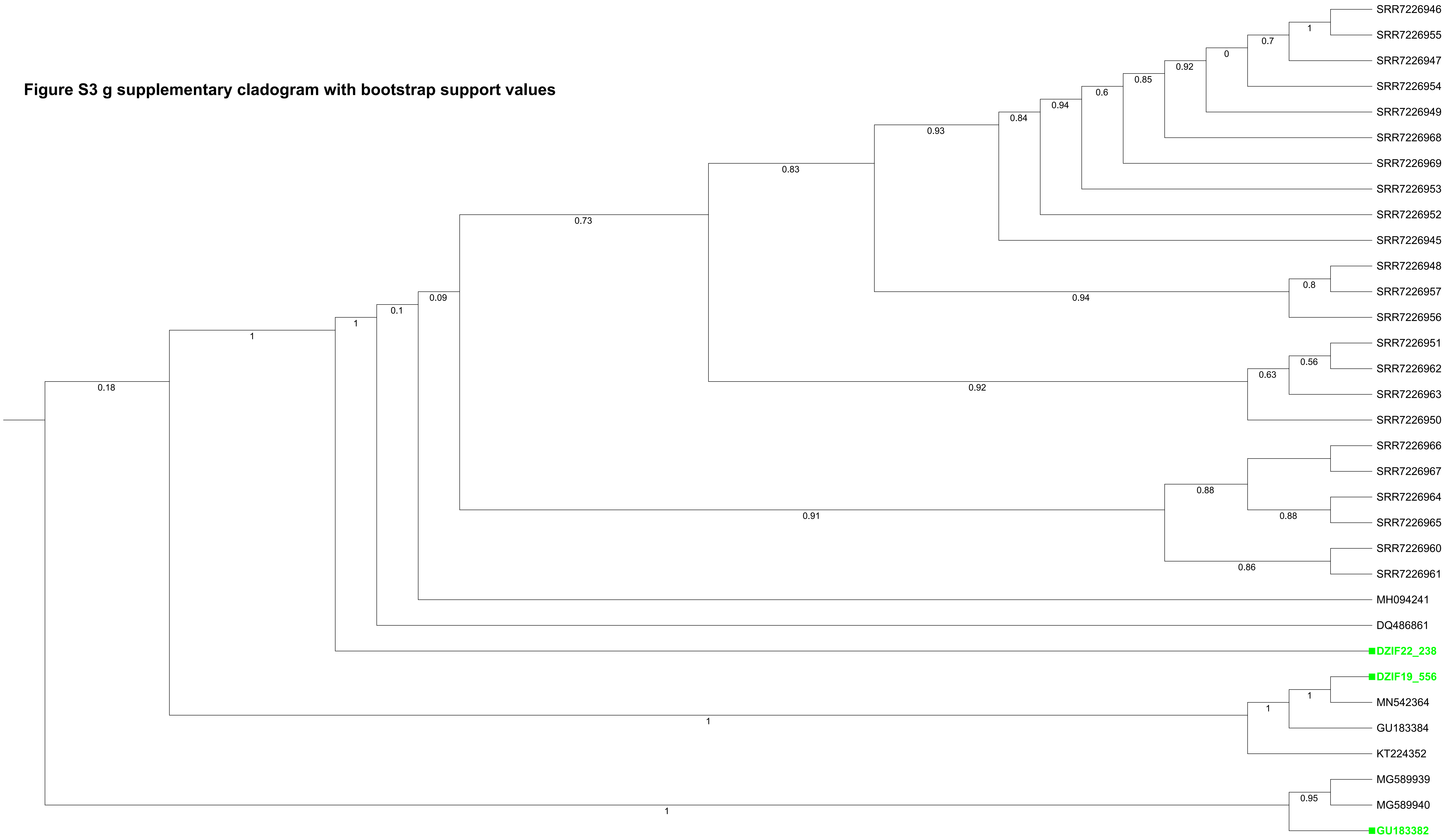

Supplement: Supplementary file 1 [file pathogens-14-00950-s001.zip › Supplementary_Figures S3.pdf]
