# Supplementary figures and images for: Distribution and Genotypic Landscape of Tick-Borne Encephalitis Virus in Ticks from Latvia from 2019 to 2023
Source: Pathogens. 2025 Sep 22;14(9):950. doi: 10.3390/pathogens14090950 (PMC12472722; doi:10.3390/pathogens14090950)

*I. persulcatus*  
only

Both

*I. ricinus* only

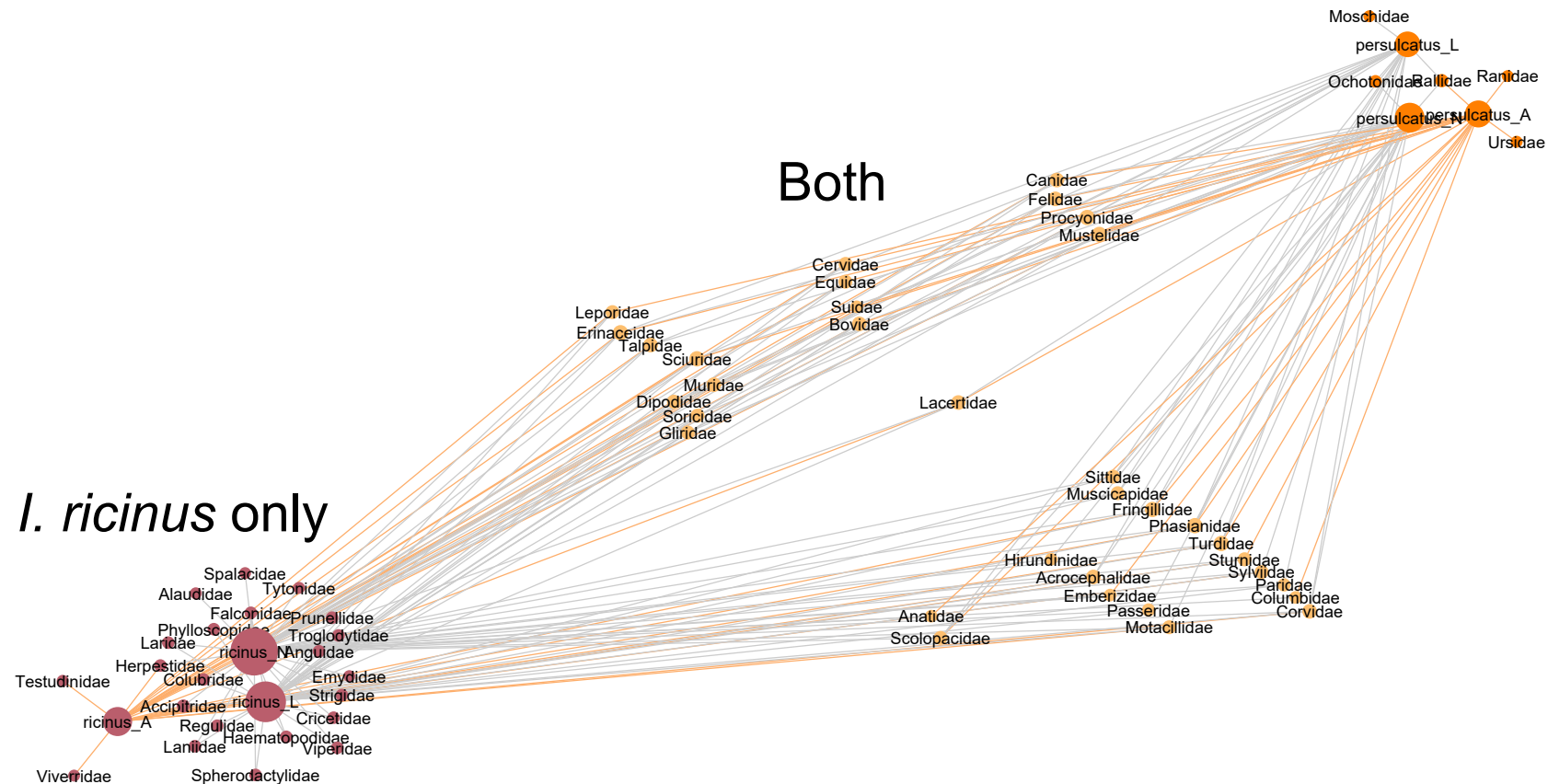

Supplement: Supplementary file 1 [file pathogens-14-00950-s001.zip › Supplementary_Figure S2.pdf]
